# Copper-67-Labeled Bombesin Peptide for Targeted Radionuclide Therapy of Prostate Cancer

**DOI:** 10.3390/ph15060728

**Published:** 2022-06-08

**Authors:** Truc T. Huynh, Ellen M. van Dam, Sreeja Sreekumar, Cedric Mpoy, Benjamin J. Blyth, Fenella Muntz, Matthew J. Harris, Buck E. Rogers

**Affiliations:** 1 Department of Radiation Oncology, Washington University School of Medicine, St. Louis, MO 63108, USA; t.huynh@wustl.edu (T.T.H.); gaadhasree@gmail.com (S.S.); ccmpoy@gmail.com (C.M.); 2 Department of Chemistry, Washington University in St. Louis, St. Louis, MO 63130, USA; 3 Clarity Pharmaceuticals Ltd., Sydney, NSW 2015, Australia; ellen.van.dam@claritypharmaceuticals.com (E.M.v.D.); matt.harris@claritypharmaceuticals.com (M.J.H.); 4 Cancer Research Division, Peter MacCallum Cancer Centre, Melbourne, VIC 3000, Australia; benjamin.blyth@petermac.org (B.J.B.); fenella.muntz@petermac.org (F.M.); 5 Sir Peter MacCallum Department of Oncology, University of Melbourne, Melbourne, VIC 3000, Australia

**Keywords:** gastrin-releasing peptide receptor, bombesin peptide antagonist, theranostic, copper-67

## Abstract

The gastrin-releasing peptide receptor (GRPR) is a promising molecular target for imaging and therapy of prostate cancer using bombesin peptides that bind to the receptor with high affinity. Targeted copper theranostics (TCTs) using copper radionuclides, ^64^Cu for imaging and ^67^Cu for therapy, offer significant advantages in the development of next-generation theranostics. [^64^Cu]Cu-SAR-BBN is in clinical development for PET imaging of GRPR-expressing cancers. This study explores the therapeutic efficacy of [^67^Cu]Cu-SAR-BBN in a pre-clinical mouse model. The peptide was radiolabeled with ^67^Cu, and specific binding of the radiolabeled peptide towards GRPR-positive PC-3 prostate cancer cells was confirmed with 52.2 ± 1.4% total bound compared to 5.8 ± 0.1% with blocking. A therapy study with [^67^Cu]Cu-SAR-BBN was conducted in mice bearing PC-3 tumors by injecting 24 MBq doses a total of six times. Tumor growth was inhibited by 93.3% compared to the control group on day 19, and median survival increased from 34.5 days for the control group to greater than 54 days for the treatment group. The ease and stability of the radiochemistry, favorable biodistribution, and the positive tumor inhibition demonstrate the suitability of this copper-based theranostic agent for clinical assessment in the treatment of cancers expressing GRPR.

## 1. Introduction

The gastrin-releasing peptide receptor (GRPR) (also known as bombesin receptor 2 (BB2)) is a seven-transmembrane receptor and is expressed on the surface of a variety of cancer cells, including breast, colon, lung, pancreatic, and prostate [1,2,3,4,5,6,7], while physiological GRPR expression in healthy tissues is low, with the exception of the pancreas [8]. Bombesin is a 14-amino acid peptide agonist that binds with high affinity to GRPR, and many analogs based on this 14-amino acid backbone have been investigated as targeting ligands for diagnosis and therapy of GRPR-positive tumors using several different radionuclides [2,9,10,11]. In addition to peptide agonists, bombesin peptide antagonists have also been evaluated in recent years [4,12,13,14]. These have generally been shown to be superior to the agonists in terms of having increased tumor uptake and fewer side effects [13]. Thus, bombesin peptide antagonists may be ideal for theranostic approaches in visualizing and treating GRPR-positive cancers [4,14].

Theranostic approaches have drawn particular interest in cancer treatment as it offers the potential to both visualize and treat diseases by incorporating both imaging and therapy within one scaffold. The ^68^Ga/^177^Lu pair has been the most widely used and has been successful in its application in targeting somatostatin receptors and prostate-specific membrane antigen (PSMA) [15,16,17]. With the recent production of the therapeutic radionuclide ^67^Cu (T_1/2_ 61.8 h, β^-^ = 100%, E_max_ = 0.577 MeV, γ = 0.185 MeV (45%)) via linear accelerators at specific activity ranging from 180–550 Ci/mg and high yields of ^64^Cu (T_1/2_ 12.7 h, β^+^ = 17.6%, β^-^ = 38.5%, EC = 43.5%, E_max_ = 0.656 MeV) from cyclotron production, there has been renewed interest in the use of ^64^Cu /^67^Cu as a theranostic pair [18,19,20,21]. Targeted copper theranostics (TCT) offers significant advantages compared to ^68^Ga/^177^Lu-based theranostics, including centralized production and broad distribution of ready-to-use TCTs for either diagnosis or therapy. This is due to the sufficient physical half-lives of both ^64^Cu and ^67^Cu and scalable product supply for ^64^Cu and ^67^Cu due to favorable production methods using cyclotrons and accelerators, respectively. Additionally, ^64^Cu can be imaged on the same day (as with current patient scheduling) but also offers the ability to collect multiple images from one hour to 48 h for flexible patient scheduling. ^67^Cu emits a gamma ray at 0.185 MeV, which is better suited for SPECT imaging, and a beta minus particle with a similar energy range and path length in tissue to ^177^Lu in addition to a shorter half-life (2.6 days) than ^177^Lu (6.7 days) makes ^67^Cu well suited to peptide pharmacokinetics and may allow more frequent administration. The longer physical half-life of ^64^Cu compared to ^68^Ga improves dosimetry estimates by PET imaging, and since ^64^Cu/^67^Cu is a “true” matched pair, the radiochemical products have the same in vitro and in vivo behavior.

**Figure 1:**
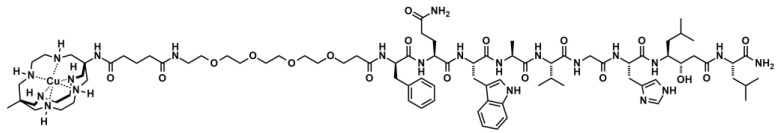
The structure of [^64/67^Cu]Cu-SAR-BBN.

The sarcophagine (MeCOSar) is a bicyclic cage-like metal chelator molecule that is well known for its strong binding of Cu^2+^, good in vivo stability, and high radiolabeling efficiency under mild conditions [22,23,24]. The metal ion is encapsulated in the ligand tightly, resulting in the formation of extraordinarily stable complexes that resist dissociation of the metal in vivo [25]. The bombesin antagonist conjugated to the sarcophagine (3,6,10,13,16,19-hexaazabicyclo[6.6.6]icosane = Sar) derivative 5-(8-methyl-3,6,10,13,16,19-hexaaza-bicyclo[6.6.6]icosan-1-ylamino)-5-oxopentanoic acid (MeCOSar) via PEG4 (SAR-BBN) has been previously evaluated after radiolabeling with ^64^Cu and demonstrated good in vitro binding and high PC-3 tumor uptake in mice [14] (Figure 1). These data prompted the clinical translation of [^6^^4^Cu]Cu-SAR-BBN, which is in development for PET imaging in breast cancer (ACTRN12619001383156) and planned studies in prostate cancer. This paper describes the radiolabeling, in vitro evaluation, biodistribution, and therapeutic efficacy of [^67^Cu]Cu-SAR-BBN in the context of prostate cancer.

## 2. Results

### 2.1. Radiochemistry

SAR-BBN was radiolabeled with [^67^Cu]CuCl_2_ and achieved > 95% radiochemical purity at 40 °C for 20 min without further purification by radio-HPLC (Figure S1) and radio-TLC (Figure S2). The molar activity for [^67^Cu]Cu-SAR-BBN was typically 30 MBq/nmol. Radio-HPLC chromatograms showed a retention time of 9.03 min for [^67^Cu]Cu-SAR-BBN (Figure S1).

### 2.2. Serum Stability

After 96 h in PBS 1X or human serum at 37 °C, [^67^Cu]Cu-SAR-BBN remained greater than 95% intact (Figure S3).

### 2.3. Cell Binding and Internalization

**Figure 2:**
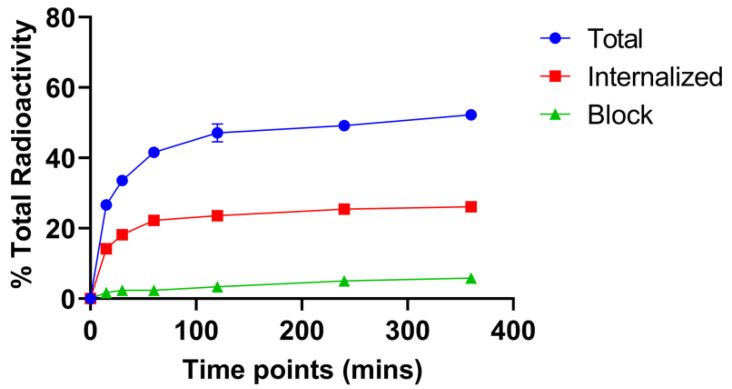
Representative binding and internalization curves of [^67^Cu]Cu-SAR-BBN. Total bound and internalization levels were shown as a percentage of the total radioactivity added. Data are presented as triplicates of mean ± SD.

Figure 2 shows in vitro uptake of [^67^Cu]Cu-SAR-BBN into the GRPR-positive PC-3 cell line. [^67^Cu]Cu-SAR-BBN accumulated into the cells at the level of 26.6 ± 1.3% at 15 min and increased up to 52.2 ± 1.4% at 6 h. Less than 30% of the total added radioactivity was internalized into the cells at 6 h. To confirm the specific binding, an excess of Tyr^4^-BBN was used to perform inhibition experiments, and the results indicated that non-specific binding only amounts to 6% of total added radioactivity.

### 2.4. Biodistribution Studies

**Figure 3:**
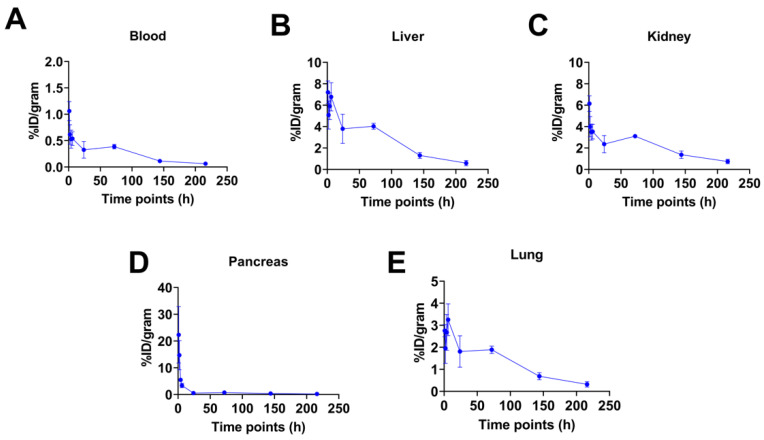
Clearance of [^67^Cu]Cu-SAR-BBN in selected tissues for C57BL/6 female mice up to 9 days (*n* = 5). After intravenous injection with 0.37 MBq radiotracers via tail vein, mice were sacrificed at 1 h, 2 h, 4 h, 6 h, 24 h, 3 days, 6 days, and 9 days. Organs were harvested and counted for activity. The plots focus on the clearance of radiotracer in selected tissues such as blood (**A**), liver (**B**), kidney (**C**), pancreas (**D**), and lung (**E**). Data are expressed as mean ± SD (% ID/g).

Figure 3 shows biodistribution profiles of [^67^Cu]Cu-SAR-BBN in selected organs evaluated over 9 days for C57BL/6 female mice. Table S1 details all biodistribution results for normal organs. Effective blood clearance was demonstrated at 1 h (1.06 ± 0.18% ID/g). At 24 h, activity circulation in the blood had reduced by over 70% of the 1 h value. The expression of GRPR in the pancreas resulted in high initial pancreas uptake, with the highest accumulated activity at 1 h (22.32 ± 10.54% ID/g), followed by rapid clearance with a 75% reduction in radioactivity by 4 h and a 97% reduction by 24 h. [^67^Cu]Cu-SAR-BBN showed low uptake in the kidneys at 1 h (6.15 ± 0.73% ID/g), and further reduction at subsequent time points suggests a rapid renal clearance. Liver uptake peaks at 1 h (7.21 ± 1.05% ID/g), suggesting hepatobiliary clearance of [^67^Cu]Cu-SAR-BBN. Low uptake was observed in the lung (2.75 ± 0.28% ID/g at 1 h), with a similar clearance pattern to that of the liver.

### 2.5. Therapy Studies

**Figure 4:**
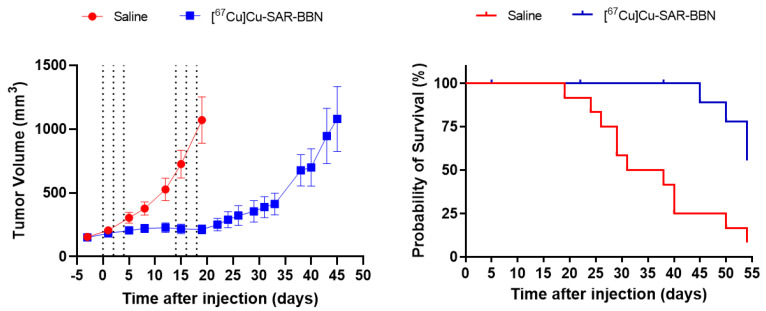
Inhibition of PC-3 tumor growth and Kaplan–Meier survival curve following treatment with 144 MBq [^67^Cu]Cu-SAR-BBN over 3 weeks, expressed as mean tumor volume (±SEM) (*n* = 12). On the left, the graph depicts average tumor volumes measured three times a week with the dotted lines indicating administration of either saline or 24 MBq [^67^Cu]Cu-SAR-BBN on day 0, 2, 4, 14, 16, and 18 of the experiment. Tumor volume was calculated as length (mm) × width (mm) × width (mm)/2. The curves illustrate tumor measurements up to the last day of all mice alive for the saline (day 19) and [^67^Cu]Cu-SAR-BBN (day 45). The right graph shows the survival curves of the saline (red) and [^67^Cu]Cu-SAR-BBN groups as mice were euthanized due to tumor exceeding the volume of 2500 mm^3^.

Administration of six doses of 24 MBq of [^67^Cu]Cu-SAR-BBN effectively inhibited tumor growth with a 93.3% reduction in tumor volume on day 19 compared to control (Figure 4). [^67^Cu]Cu-SAR-BBN significantly prolonged survival, defined as survival to tumor endpoint of 2500 mm^3^ (median survival 34.5 vs. >54 days; *p* = 0.0015). There was no significant weight loss in mice from both groups during the course of the study (Figure S4). However, one mouse from the [^67^Cu]Cu-SAR-BBN group was euthanized due to tumor ulceration (day 38), and another two died due to unknown causes (day 5 and day 22). As the deaths of these three mice were not related to tumor volume, they were censored in the survival analysis.

### 2.6. Immunohistochemical Staining Assay

**Figure 5:**
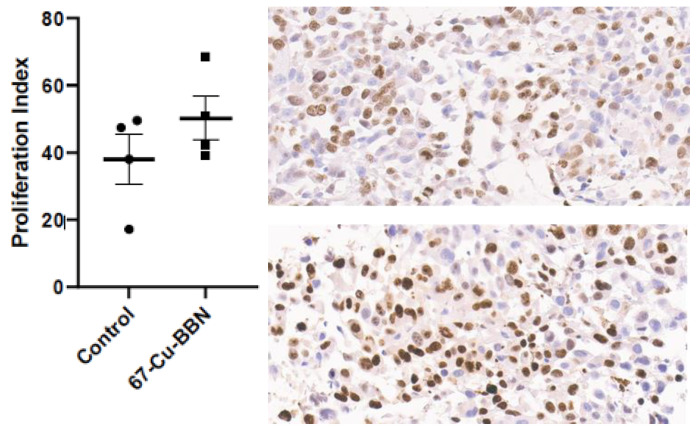
Proliferation index in areas of tumors without necrosis, immune infiltrate or host mouse stroma. The left graph depicts PI values from 4 tumors collected from either saline (control) or 144 MBq [^67^Cu]Cu-SAR-BBN group. On the right, a fixed field (20× magnification) was used to identify a region of interest (ROI) containing proliferating tumor cells (not stroma, necrosis, or immune infiltrate). The tumor from mouse 48—control group and tumor from mouse 44—[^67^Cu]Cu-SAR-BBN group are shown in the top and bottom panels, respectively.

With regards to kidney, pancreas, and liver, no significant histological changes were observed between the control and [^67^Cu]Cu-SAR-BBN treated mice (Figure S5). A blinded review of tumor samples revealed that all tumors contained some areas of necrosis, but [^67^Cu]Cu-SAR-BBN treated mice were identified as having more extensive areas of necrosis in three out of four tumors (Figure S6). Initial examination of the Ki-67-stained tumor tissues revealed that treated tumors contained large areas of complete necrosis with no tumor cells present. In the areas of tumors that showed no necrosis, immune infiltrate or host mouse stroma, the proliferation index (PI) across tumors ranged from 17 to 69%, with no significant difference between the treated and control groups. Specifically, the mean PI was 38.1% and 50.3% in control and [^67^Cu]Cu-SAR-BBN groups, respectively (*p* = 0.26, unpaired *t*-test, two-tailed) (Figure 5). Estimation of PI was hindered by spatial heterogeneity between the necrosis at the core and periphery of each tumor and limited areas of proliferating tumors (not stroma, necrosis, or immune infiltrate), so PI may not be representative of the overall health or proliferation of the tumors.

## 3. Discussion

Throughout the years, many antagonistic and agonistic radiolabeled bombesin derivatives have been assessed for the imaging of GRPR-expressing tumors [2,3,4]. Kahkonen et al. reported the first in-human study of ^68^Ga-bombesin antagonist BAY867548, in which PET imaging successfully detected all dominant lesions with an accuracy of 83% for detection of the organ-confined disease and 70% sensitivity in the detection of lymph node metastases in human subjects with histologically confirmed prostate adenocarcinoma [26]. ^18^F-labeled bombesin analog BAY864367 was also evaluated in patients with prostate cancer and showed favorable dosimetric values with the average tumor-to-background ratio of 12.9 ± 7.0 and mean effective dose of 4.3 ± 0.3 mSv/patient [27]. In another clinical study, a bombesin-derived RP527 radiolabeled with ^99m^Tc has shown specific tumor localization with good tumor-to-normal-tissue ratios in patients with metastasized prostate or breast carcinoma [28]. Despite promising results of radiolabeled BBN derivatives as tumor imaging probes, there are a limited number of therapeutic studies. Researchers have performed a pre-clinical evaluation of bombesin derivatives labeled with ^90^Y, ^177^Lu, and ^188^Re for the targeted radiotherapy of prostate cancer, which demonstrated good tumor targeting and therapeutic efficacy in mouse models [17,29,30,31,32]. Of these, ^177^Lu has been the most attractive as a theranostic agent because of its favorable β-energy and pairing with ^68^Ga for imaging. However, the ^68^Ga/^177^Lu pair do have some shortcomings owing to the short half-life of ^68^Ga (68 min) that does not match with the physical half-life of ^177^Lu (6.7 days), and the longer half-life of ^177^Lu not suited to the pharmacokinetics of peptide-based drugs. Additionally, the pair is not of the same element, and slight chemical differences between the two may lead to differences in the biological behavior of radiolabeled complexes in vivo.

Therefore, to obviate these problems, we chose TCTs based on ^67^Cu (E_max_ = 0.562 MeV) as a therapeutic radionuclide because it has an optimal β-energy (E_max_ = 0.577 MeV) and, therefore, path length to provide irradiation of the tumor mass while preserving surrounding healthy tissue, and can be paired with ^64^Cu for imaging. In this regard, ^64^Cu-labeled SAR-BBN has been evaluated in the PC-3 tumor mouse model by biodistribution as well as PET imaging [14]. Biodistribution studies of [^64^Cu]Cu-SAR-BBN demonstrated a PC-3 tumor uptake of 19.6 ± 4.7% ID/g at 1 h as compared to 0.9 ± 0.1% ID/g in the presence of excess blocking, which is the highest radioactivity accumulation among all investigated organs [14]. High tumor-to-normal-tissues ratios at 1, 4, and 24 h showed selective uptake of [^64^Cu]Cu-SAR-BBN to GRPR expressing PC-3 tumors as compared to other tissues such as muscle, liver, and kidney [14]. PET images also revealed radioactivity accumulation in GRPR-positive organs such as tumors and the pancreas with only marginal uptake in other tissues [14]. These results, along with sufficient safety data, enabled translation to an Australian-based clinical trial in breast cancer (ACTRN12619001383156).

Here we focus on the radiolabeling and pre-clinical evaluation of [^67^Cu]Cu-SAR-BBN. The molar activity of [^67^Cu]Cu-SAR-BBN was 30 MBq/nmol, three-fold lower than the previously reported 100 MBq/nmol for [^64^Cu]Cu-SAR-BBN but still within the range of 12 MBq/nmol for GRPR-analogues coupled with various SAR-derived chelators [14,33]. Human serum stability of [^67^Cu]Cu-SAR-BBN showed <5% free ^67^Cu at 96 h. Additionally, in vitro cell studies of [^67^Cu]Cu-SAR-BBN revealed a gradual increase in cellular uptake over time following continued exposure of PC-3 cells to [^67^Cu]Cu-SAR-BBN, which is similar to results reported by Gourni et al. [14]. At 37 °C, more than 50% of [^67^Cu]Cu-SAR-BBN was bound to the GRPR receptors, and about 20% internalized into the cells, which is comparable to the results reported for [^64^Cu]Cu-SAR-BBN with 60% bound to the cells and 20% internalized [14]. Biodistribution studies with C57BL/6 female mice showed rapid clearance of the radiolabeled peptide in the blood and normal tissues such as kidney and liver. Initial high radioactivity uptake was detected in GRPR-positive pancreas but was quickly cleared at subsequent time points. The effective clearance from non-target organs highlighted the potential of [^67^Cu]Cu-SAR-BBN as a therapeutic agent due to limited normal tissue toxicity.

In this paper, the therapy study was performed in PC-3 tumor-bearing mice administered with 6 × 24 MBq [^67^Cu]Cu-SAR-BBN on day 0, 2, and 4, a drug-free week followed by another three injections on day 14, 16, and 18. The total dose of 144 MBq was derived from the literature in which one group of mice with subcutaneous PC-3 tumors were treated with the same fractionated dosing of 144 MBq [^177^Lu]Lu-RM2—another GRPR antagonist derived from bombesin analogs [34]. Unfortunately, tumor volumes were not reported in this manuscript, and they reported that 4/10 animals experienced a rapid recurrence of the tumor and were sacrificed, while 6/10 showed complete remission out to 150 days. In addition, when they were treated with a total of 72 MBq over six doses, survival results were similar. Our therapy with [^67^Cu]Cu-SAR-BBN showed a 93.3% tumor inhibition at day 19 compared to the control group, with the treated mice demonstrating tumor growth after this time. Survival curves show that the median survival for our control group was 34.5 days compared to more than 54 days for mice treated with [^67^Cu]Cu-SAR-BBN. Compared to [^177^Lu]Lu-RM2 in the Dumont et al. paper, which had 40% of the treated animals with rapid recurrence of the tumors and sacrificed early, [^67^Cu]Cu-SAR-BBN had a more uniform result with no animals having a rapid recurrence and needing to be sacrificed early. The therapeutic dosing regimen can still be optimized by potentially determining the number of doses needed to achieve a prolonged therapeutic effect and/or determining the intervals between doses, as the shorter half-life of ^67^Cu compared to ^177^Lu may allow for shorter treatment intervals. For example, a recent paper on [^177^Lu]Lu-NeoB in animal models has proposed a weekly dose of 37 MBq for 3 weeks, which resulted in tumor inhibition in gastrointestinal stromal tumors (GIST) xenograft model [35].

Treatment-related toxicity for [^67^Cu]Cu-SAR-BBN was evaluated based on body weight loss and blinded histologic examination of kidneys, pancreas, livers, and tumors of four mice from each group. There was no evidence of significant weight loss, which demonstrated that ^67^Cu does not introduce noticeable toxicity. Dumont et al. revealed that histological samples of tumors, kidneys, and pancreas collected from mice treated with 144 MBq [^177^Lu]Lu-RM2 showed no abnormalities as compared to the control mice [34]. Similarly favorable observations were made for those organs obtained from mice treated with [^67^Cu]Cu-SAR-BBN in our study. With regards to Ki-67 immunohistochemistry staining, scoring of PI revealed similar levels of proliferation in tumor regions that showed no necrosis, immune infiltrate, or host mouse stroma for the control and [^67^Cu]Cu-SAR-BBN treated group. However, this finding is inconclusive due to significant variation between different spatial areas of any one tumor.

In summary, the PC-3 cell-based studies reflect the specific uptake of [^67^Cu]Cu-SAR-BBN in GRPR-positive cell lines. Biodistribution results show marginal uptake in nontumor tissues after 4 h, while therapy and immunohistology studies reveal tumor growth inhibition and limited toxicity to non-target organs. Thus, the bombesin antagonist peptide [^67^Cu]Cu-SAR-BBN may be a suitable theranostic approach for treating a variety of GRPR-positive cancers.

## 4. Materials and Methods

### 4.1. General Information

All solvents and reagents were purchased from Sigma-Aldrich (St. Louis, MO, USA) or Fisher Scientific (Pittsburgh, PA, USA) and used as received unless stated otherwise. All solutions and buffers were prepared using HPLC-grade water. Radio-TLCs employed Whatman 60 Å silica gel thin-layer chromatography (TLC) plates and were analyzed using a Bioscan 200 imaging scanner (Bioscan, Inc., Washington, DC, USA). Radioactivity was counted with a Beckman Gamma 8000 counter containing a NaI crystal (Beckman Instruments, Inc., Irvine, CA, USA). Reversed-phase high-pressure liquid chromatography (HPLC) was used to evaluate the radiolabeling efficiency. HPLC utilizes a two-solvent system: water (0.05% trifluoroacetic acid (TFA)) and acetonitrile (0.05% TFA). The system was equipped with UV absorbance detectors (UV, 220 nm and 280 nm), a NaI radiotracer detector, and a photodiode array detector. HPLC analysis of peptides used Kinetex (Phenomenex, Torrance, CA, USA) C-18 column (5 μm, 4.6 × 150 mm I.D.).

SAR-BBN was synthesized and characterized by Auspep Pty Ltd. (Melbourne, Australia). SAR-BBN was diluted in HPLC-grade water to make a stock solution of 1 nmol/10 μL. The bombesin analog Tyr^4^-BBN (pGlu-Gln-Arg-Tyr-Gly-Asn-Gln-Trp-Ala-Val-Gly-His-Leu-Met-NH_2_) was used as a blocking agent and purchased from Sigma-Aldrich (St. Louis, MO, USA).

### 4.2. Cell Culture

The PC-3 cell line was purchased from ATCC (Manassas, VA, USA), revived from liquid nitrogen storage, and cultured at 37 °C, 5% CO_2_. PC-3 cells were cultured in media containing DMEM, 10% heat-inactivated fetal bovine serum (FBS) (Gibco, Thermo Fisher Scientific, Inc., Waltham, MA, USA), and 10 mM HEPES.

### 4.3. Radiochemistry

[^67^Cu]CuCl_2_ was obtained from the Idaho Accelerator Center (Pocatello, ID, USA). For radiolabelling, 1.0 nmol of SAR-BBN (10 μL by volume) was added to 0.8 mCi (30 MBq) of [^67^Cu]CuCl_2_ in 100 µL of 0.4 M NH_4_Oac (pH = 5.5) and incubated at 40 °C for 20 min. Radio-HPLC analysis was performed with a mobile phase of water (0.1% TFA) and acetonitrile (0.1% TFA), 5−90% acetonitrile in 10:30 min, and elution was run with a 1 mL/min flow rate. Radio-TLCs with a mobile phase of 50 mM diethylenetriamine pentaacetate (DTPA) were performed to evaluate radiolabeling efficiency. For radio-TLCs, the radiolabeled complex remained at the origin, while the free ^67^Cu moved with the solvent front. No further purification was needed if radiochemical yield of greater than 95% was achieved. For the therapy study, 10 nmol of SAR-BBN was added to 8.0 mCi (300 MBq) of ^67^Cu, and the resulting product was evaluated for radiolabeling efficiency using radio-TLCs.

### 4.4. Serum Stability

Briefly, 100 µL of the radiolabeled complex was added to 100 µL PBS 1X or human serum (HSA 1 g/mL) in triplicate. [^67^Cu]Cu-SAR-BBN was incubated at 37 °C for 14, 24, 48, 72, and 96 h with moderate agitation. An aliquot (0.5 μL) was withdrawn at each specified time point. Radio-TLCs with a mobile phase of 50 mM DTPA were performed to evaluate the percentage of intact radiolabeled peptides.

### 4.5. Cell Binding and Internalization

A PC-3 cell suspension was prepared in 1X PBS at a concentration of 10^6^ cells/100 μL. Eppendorf tubes (in triplicates) were pretreated with 5% bovine serum albumin (BSA) in PBS to block non-specific binding. Tyr^4^-BBN, as a blocking agent, was diluted in 5% BSA in PBS. Then, 100 μL of cells and either 20 μg block or PBS 1X were added to each tube. Approximately 100,000 cpm of [^67^Cu]Cu-SAR-BBN was added to the tubes. Cells were incubated at 37 °C at moderate speed to prevent cell settling. After each specified time point (30, 60, 120, 240, and 360 min), cells were centrifuged and the supernatant was aspirated from cell pellets, which was followed by two additional rinses with ice-cold PBS. Cells pellets were then collected and counted for activity on a gamma counter. To determine the fraction of internalized radiotracer, cells were treated with 2 × 0.5 mL of 20 mM sodium acetate (pH 4.0) for 2 min to remove surface-bound radioactivity and counted again for activity.

### 4.6. Biodistribution Studies

To determine the biodistribution of [^67^Cu]Cu-SAR-BBN in normal tissues, 40 female C57BL/6 mice were evaluated in groups of 5 mice at various time points. [^67^Cu]Cu-SAR-BBN was prepared in 0.9% saline with a concentration of 0.37 MBq (10 µCi) per 100 µL. Mice (*n* = 5) were injected with 0.37 MBq radiotracers via tail vein and sacrificed by cervical dislocation at 1 h, 2 h, 4 h, 6 h, 24 h, 3 days, 6 days, and 9 days. Blood, lung, liver, spleen, kidney, muscle, heart, bone, and pancreas were harvested and counted for activity on a gamma counter. The percent injected dose per gram for each tissue of interest (% ID/g) was calculated and expressed as Mean ± SD. All radioactivity measurements were corrected for decay.

### 4.7. Therapy Studies

To assess the therapeutic potential of [^67^Cu]Cu-SAR-BBN, athymic nude mice bearing subcutaneous PC-3 tumors with a tumor size of ~150 mm^3^ were randomly assigned into 2 groups (*n* = 12 mice per group). For treatment, mice were dosed intravenously with three injections of 24 MBq [^67^Cu]Cu-SAR-BBN on days 0, 2, and 4, followed by a drug-free week before the injections of 24 MBq on days 14, 16, and 18. The total activity injected corresponded to 144 MBq. The control group was injected via tail vein with six injections of 0.9% saline at the same time points. Body weight and tumor volume (through caliper measurements) were monitored and recorded three times a week. Tumor size was calculated by the following formula: L × W^2^/2, where L is the long diameter of the tumor and W is the short diameter of the tumor. Mice were euthanized when the tumor size exceeded the volume of 2500 mm^3^, body weight dropped by more than 20%, or ulcerations > 4 mm. Any remaining mice at day 54 were euthanized, and tumor volume and survival curves were generated and analyzed to evaluate the efficacy of [^67^Cu]Cu-SAR-BBN. Percentage tumor growth inhibition was calculated as 100 × (1 − ΔT/ΔC), where ΔC and ΔT were determined by subtracting the mean tumor volume (in the vehicle control and treated group, respectively) on day 0 of treatment from the mean tumor volume on day 19 (the last day all mice remained in the study). Survival was defined as the time for a tumor to reach a volume of ≥2500 mm^3^.

### 4.8. Immunohistochemical Staining Assay

The kidneys, pancreas, livers, and tumors of 4 control mice and 4 treated mice were harvested and fixed in neutral buffered formalin. After fixation and dehydration, tissue samples were embedded in paraffin, and tissue sections were then stained with either hematoxylin and eosin (H&E) or Ki-67 for microscopic examination. H&E staining of tissue samples was prepared by the Anatomic and Molecular Pathology Core Lab, Washington University in St Louis. Ki-67 immunostaining was performed on 5 µm tissue sections of PC-3 tumors. The slides were baked in 55 °C oven for 60 min. The sections were deparaffinized, and heat-induced antigen retrieval was performed in citrate buffer 0.1 M pH 6.0 at 92 °C. After 20 min, the slides were cooled in 4 °C fridge for 15 min. The sections were then blocked with Dako Endogenous Enzyme Block (Dako North America, Inc., Carpinteria, CA, USA) for 10 min and washed twice with Dako Wash Buffer 1X. The sections were blocked with 10% goat serum in PBS for another 45 min and washed 2 × 5 min with Dako Wash Buffer 1X. The slides were then rinsed and incubated with Ki-67 antibody (1:100 dilution) overnight at 4 °C. The next day, ImmPRESS Goat Anti-rabbit secondary antibody (Vector Laboratories, Inc., Burlingame, CA, USA) was added to the slides for 45 min, followed by DAB and Chromogen (1:1) (Dako) for color development. The sections were rinsed and counterstained with Hematoxyline and dehydrated with alcohol and xylene, and cover-slipped. Staining results were assessed by Olympus fluorescent microscope (BX51) and the cell Sense software in a blinded manner.

### 4.9. Statistical Analysis

Data were processed using Prism 9 (GraphPad Software, v 6.03, La Jolla, CA, USA), and statistical analysis for survival was performed using a Mantel–Cox log-rank test. A *p* < 0.05 was considered statistically significant.

## 5. Conclusions

In conclusion, [^67^Cu]Cu-SAR-BBN exhibited high specific uptake in GRPR-expressing PC-3 cells in vitro. Treatment with a six-dose regimen of 144 MBq [^67^Cu]Cu-SAR-BBN had no noticeable toxicity and resulted in significant tumor growth inhibition and prolonged survival rate in PC-3 tumor-bearing mice. A biodistribution study suggests rapid renal clearance of [^67^Cu]Cu-SAR-BBN. These data demonstrate the efficacy of ^67^Cu-labeled SAR-BBN in an animal model and, combined with human data currently being generated on [^64^Cu]Cu-SAR-BBN, has promising potential as a theranostic pair for clinical translation in cancers expressing GRPR such as breast cancer, glioblastoma, and prostate cancer. The study provides further support for the use of TCTs as the ideal pairing for next-generation radiopharmaceutical theranostics.

## Data Availability

Data is contained within the article and supplementary material.

## Supplementary Materials

The following supporting information can be downloaded at: https://www.mdpi.com/article/10.3390/ph15060728/s1, Figure S1: HPLC chromatograms showing the [^67^Cu]Cu-SAR-BBN; Figure S2: Radio-TLC chromatograms of [^67^Cu]Cu-SAR-BBN in 50mM DTPA; Figure S3: Stability assays of [^67^Cu]Cu-SAR-BBN; Figure S4: Body weight changes of PC-3 tumor-bearing mice injected with either saline (as control) or [^67^Cu]Cu-SAR-BBN (*n* = 9 or 12, bars SEM); Figure S5: Hematoxylin–Eosin (H&E) staining of kidneys, livers, and pancreas tissue slices with (A) Saline (as control) (B) [^67^Cu]Cu-SAR-BBN; Figure S6: Proliferation heatmap of (A) Tumor #48C, Control (B) Tumor #41, [^67^Cu]Cu-SAR-BBN; Table S1: Biodistribution report of [^67^Cu]Cu-SAR-BBN in C57BL/6 female mice bearing GRPR-positive PC-3 xenograft tumors.
