# Supplementary material for: Copper-67-Labeled Bombesin Peptide for Targeted Radionuclide Therapy of Prostate Cancer
Source: Pharmaceuticals (Basel). 2022 Jun 8;15(6):728. doi: 10.3390/ph15060728 (PMC9229378; doi:10.3390/ph15060728)
Supplement: Supplementary file 1 [file pharmaceuticals-15-00728-s001.zip › pharmaceuticals-1727833-supplementary.pdf]

Supplementary Information for

Copper-67 Labeled Bombesin Peptide for Targeted Radiotherapy of Prostate Cancer

Truc T. Huynh, Ellen M. van Dam, Sreeja Sreekumar, Cedric Mpoy, Benjamin J. Blyth, Fenella Muntz, Matthew J. Harris, and Buck E. Rogers

TABLE OF CONTENTS	Page
Figure S1: HPLC chromatograms showing the [^{67}Cu]Cu-SAR-BBN	2
Figure S2: Radio-TLC chromatograms of [^{67}Cu]Cu-SAR-BBN in 50mM DTPA	3
Figure S3: Stability assays of [^{67}Cu]Cu-SAR-BBN	4
Figure S4: Body weight changes of PC-3 tumor-bearing mice injected with either saline (as control) or [^{67}Cu]Cu-SAR-BBN (n = 12, bars SEM)	5
Figure S5: Hematoxylin-Eosin (H&E) staining of kidneys, livers, and pancreas tissue slices with (A) Saline (as control) (B) [^{67}Cu]Cu-SAR-BBN	6
Figure S6: Proliferation heatmap of (A) Tumor #48C, Control (B) Tumor #41, [^{67}Cu]Cu-SAR-BBN	7
Table S1: Biodistribution report of [^{67}Cu]Cu-SAR-BBN in C57BL/6 female mice	8

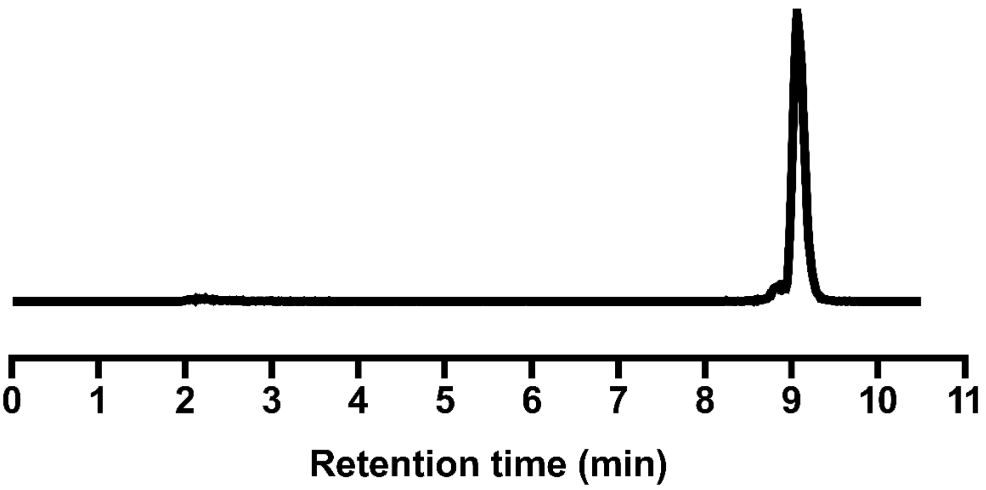

Figure S1: HPLC chromatograms showing the [^{67}Cu]Cu-SAR-BBN. Radio-HPLC analysis was performed with a mobile phase of water (0.1% TFA) and acetonitrile (0.1% TFA), 5–90% acetonitrile in 10:30 min, and elution was run with a 1 mL/min flow rate.

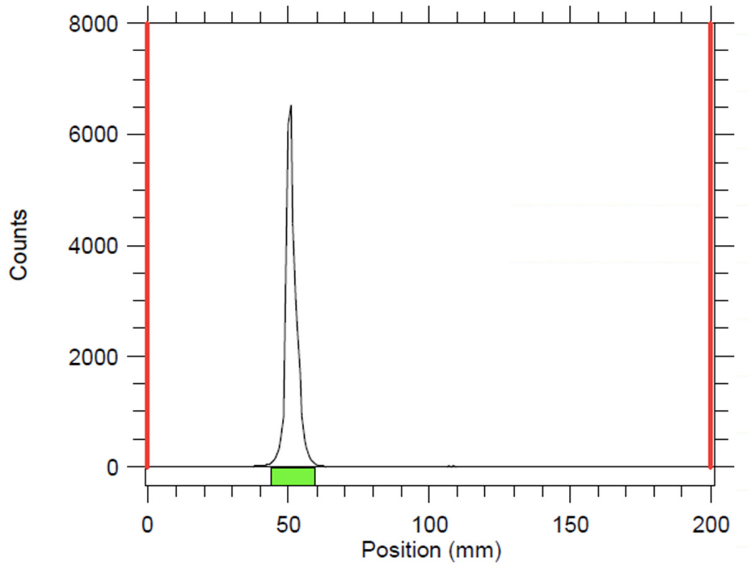

Figure S2: Radio-TLC chromatograms of [^{67}Cu]Cu-SAR-BBN in 50mM DTPA .

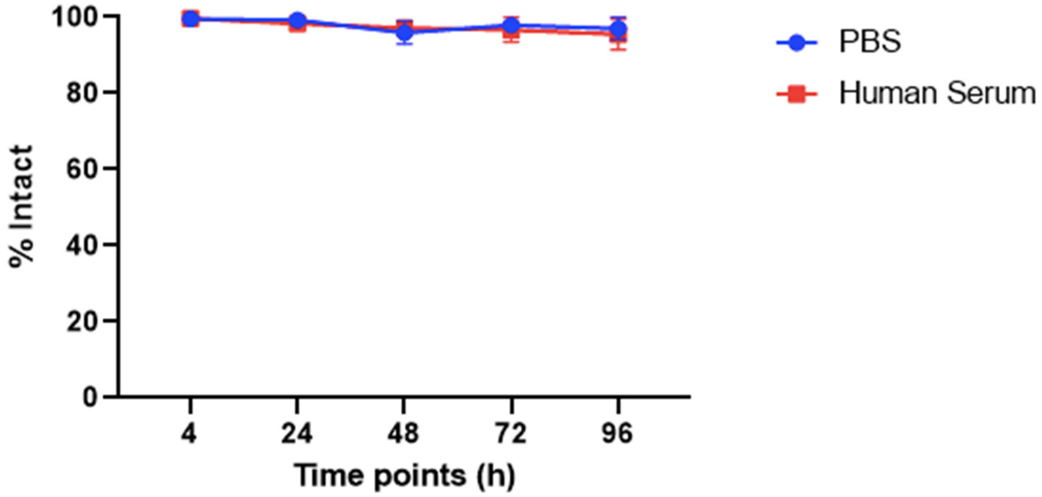

Figure S3: Stability assays of [^{67}Cu]Cu-SAR-BBN.

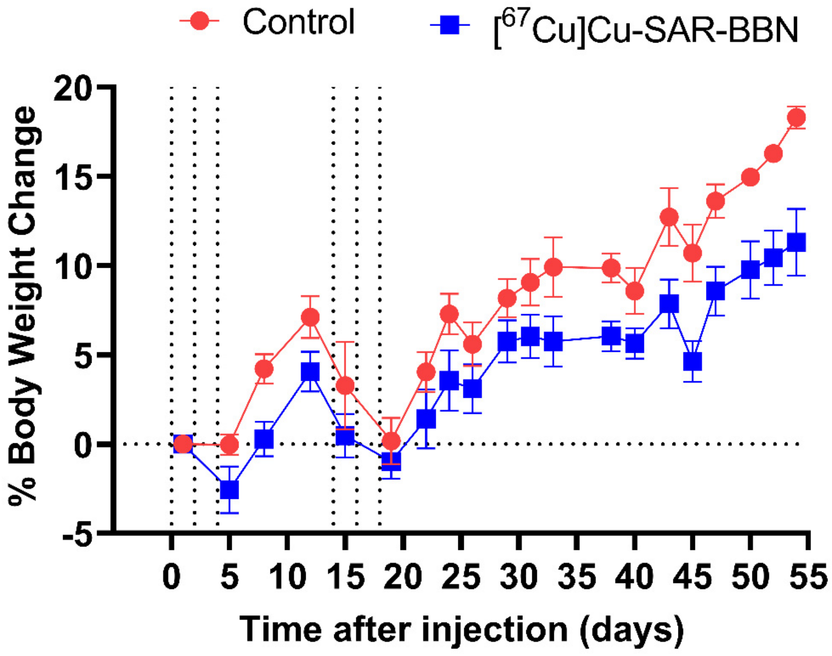

Figure S4: Body weight changes of PC-3 tumor-bearing mice injected with either saline (as control) or [^{67}Cu]Cu-SAR-BBN (n = 12, bars SEM). The doses were given on days indicated by dotted lines via tail vein. The data represents the mean percent weight change from baseline (day 1) for each group.

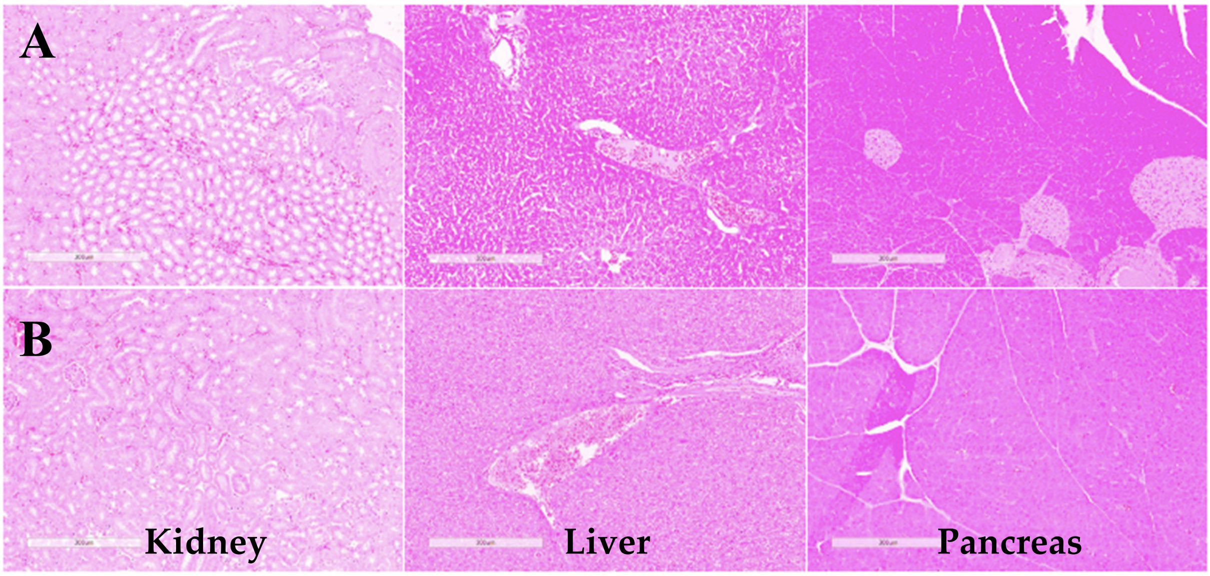

Figure S5: Hematoxylin-Eosin (H&E) staining of kidneys, livers, and pancreas tissue slices with (A) Saline (as control) (B) [^{67}Cu]Cu-SAR-BBN.

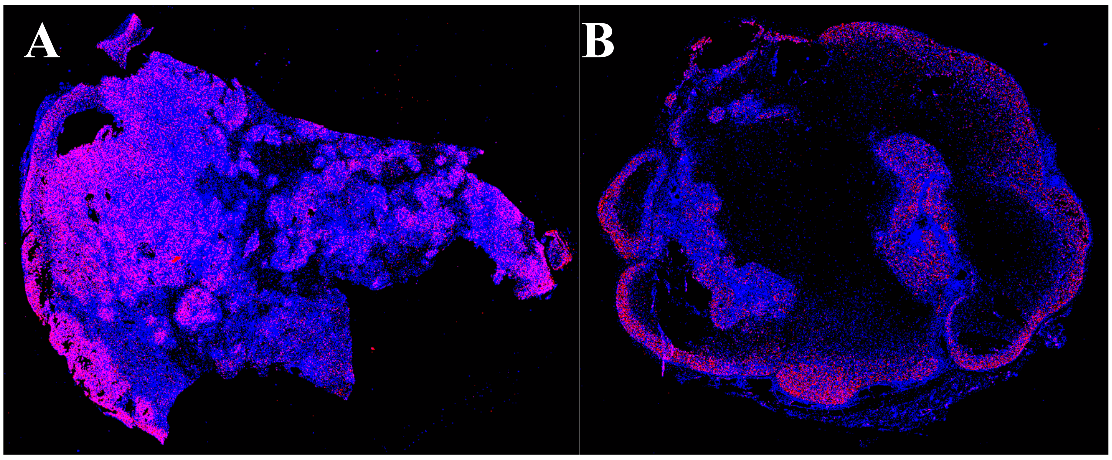

Figure S6: Proliferation heatmap of (A) Tumor #48C, Control (B) Tumor #41, [^{67}Cu]Cu-SAR-BBN. Control group tumors contain limited areas of poor cellularity/necrosis (black zones), large areas of tumor with moderate proliferation (sparse red/blue signal) and high proliferation areas at the periphery (intense red signal). [^{67}Cu]Cu-SAR-BBN treated tumors contain extensive areas of necrosis (black zones), limited areas of surviving tumor with limited proliferation (sparse red/blue signal) and high proliferation areas at the periphery (intense red signal).

Table S1: Biodistribution report of [^{67}Cu]Cu-SAR-BBN in C57BL/6 female mice.

	1 h		2 h		4 h		6 h	
	**%ID/g**	**SD**	**%ID/g**	**SD**	**%ID/g**	**SD**	**%ID/g**	**SD**
**Blood**	1.06	0.18	0.62	0.19	0.53	0.17	0.54	0.13
**Lung**	2.75	0.28	1.95	0.68	2.68	0.81	3.25	0.72
**Liver**	7.21	1.05	5.08	1.31	5.90	1.25	6.78	1.31
**Spleen**	1.94	0.72	1.27	0.37	0.94	0.15	0.90	0.12
**Kidney**	6.15	0.73	3.99	0.95	3.48	0.75	3.53	0.68
**Muscle**	0.52	0.17	0.25	0.10	0.35	0.07	0.59	0.63
**Bone**	0.88	0.14	0.40	0.12	0.47	0.12	0.46	0.14
**Heart**	1.00	0.10	0.58	0.21	0.78	0.28	0.84	0.22
**Pancreas**	22.32	10.54	14.71	5.43	5.48	0.73	3.38	0.80
	**24 h**		**72 h**		**6 days**		**9 days**	
	**%ID/g**	**SD**	**%ID/g**	**SD**	**%ID/g**	**SD**	**%ID/g**	**SD**
**Blood**	0.33	0.16	0.39	0.04	0.11	0.03	0.06	0.03
**Lung**	1.81	0.72	1.89	0.17	0.68	0.16	0.32	0.12
**Liver**	3.80	1.36	4.03	0.28	1.31	0.28	0.59	0.24
**Spleen**	0.51	0.20	0.74	0.08	0.30	0.07	0.14	0.05
**Kidney**	2.37	0.80	3.10	0.10	1.37	0.34	0.74	0.20
**Muscle**	0.20	0.11	0.27	0.04	0.21	0.07	0.15	0.04
**Bone**	0.31	0.22	0.25	0.16	0.09	0.03	0.09	0.03
**Heart**	0.84	0.42	1.35	0.29	0.93	0.28	0.58	0.20
**Pancreas**	0.55	0.27	0.75	0.11	0.39	0.09	0.22	0.06
